# Severe Intraoperative Bradycardia during Laparoscopic Cholecystectomy due to Rapid Peritoneal Insufflation

**DOI:** 10.1155/2020/8828914

**Published:** 2020-06-06

**Authors:** Mohammed Heyba, Ahmed Khalil, Yasser Elkenany

**Affiliations:** ^1^Kuwait Board of Anesthesiology, Kuwait City, Kuwait; ^2^Department of Anesthesia and Intensive Care, Jahra Hospital, Al Jahra, Kuwait

## Abstract

Laparoscopy is becoming increasingly popular in gynecological and general surgical operations. There are complications that are inherent to the laparoscopy techniques; amongst them is intraoperative vagal-mediated bradycardia that results from peritoneal stretching. This can occur due to high flow rate of gas during peritoneal insufflation, a practice still happening nowadays. We report a case of a middle-aged hypertensive patient who was undergoing elective laparoscopic cholecystectomy. The patient was assessed more than once preoperatively by the anesthesia team for blood pressure optimization. The patient underwent general anesthesia and developed severe bradycardia immediately after peritoneal insufflation. The management started immediately by stopping the insufflation and deflating the abdomen. Afterwards, atropine was administered intravenously, and CPR was started preemptively according to the ACLS protocol to prevent the patient from progressing into cardiac arrest. She responded to the management and became vitally stable within one minute. After confirming that there was no cardiac or metabolic insult through rapid blood investigations and agreeing that the cause of bradycardia was the rapid insufflation, the surgical team proceeded with the surgery in the same setting using low flow rate of CO_2_ to achieve pneumoperitoneum. There were no complications in the second time and the operation was completed smoothly. The patient was extubated and shifted to the postanesthesia care unit to monitor her condition. The patient was stable and conscious and later shifted to the wards and discharged on routine follow-up after confirming that there were no complications in the postoperative follow-up. Therefore, it is important to monitor the flow rate of CO_2_ during peritoneal insufflation in laparoscopic surgery as rapid peritoneal stretch can cause severe bradycardia that might progress into cardiac arrest, especially in hypertensive patients. It is also important for the anesthetist to be vigilant and ready to manage such cases.

## 1. Introduction

Laparoscopic surgery is extremely popular nowadays for emergency and elective procedures in general surgery and gynecology fields [1]. Advantages of laparoscopic surgery include decreased postoperative pain, better cosmetic outcome, improved pulmonary function, shorter hospital stay, and reduced overall cost of health services. However, there are specific complications inherent to laparoscopy, including major vascular injury, gas embolism [2], and hemodynamic instability [3]. Among the major complications of laparoscopy is vagal-mediated bradycardia, although this complication is rare. Bradycardia is defined as a heart rate (HR) below 60 beats per minute (bpm). Common causes of intraoperative bradycardia and bradycardic arrest include block of sympathetic tone by neuraxial anesthesia or drugs, vagotonic drugs, or physical stimulation that increases vagal activity, like what occurs during laparoscopy [4]. Bradycardia and cardiac arrest were first described in experimental models of laparoscopy and real cases in the early 1970s [5–7]. The first cases of intraoperative bradycardia reported were for laparoscopic gynecological surgeries [8, 9]. Later, there were a few reports of bradycardia incidents during laparoscopic general surgical procedures, including laparoscopic cholecystectomy [10, 11]; in some cases, patients progressed into cardiac arrest [10, 11]. Although there is no clear estimate of the incidence of this complication, several case series report that the incidence of bradyarrhythmia during laparoscopy in healthy individuals ranges from 14% to 30% [12–14]. Here, we report a case of a female patient undergoing laparoscopic cholecystectomy who experienced severe bradycardia approaching cardiac arrest after peritoneal insufflation, in addition to discussing the intraoperative management and postoperative course of the patient.

## 2. Case

**Figure 1:**
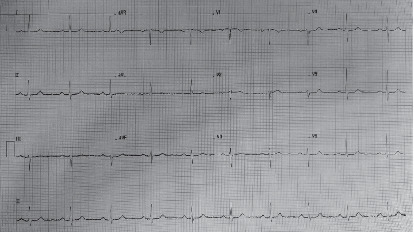
Preoperative electrocardiogram (ECG) for the patient performed in the anesthesia clinic.

A 58-year-old female patient, known to be hypertensive on medical treatment, was planned for elective laparoscopic cholecystectomy for cholelithiasis. The patient was seen in the anesthesia clinic preoperatively, where her medical history was taken, and physical examination was performed. The patient had a past medical history of essential hypertension on treatment by valsartan 160 milligrams daily and bisoprolol 5 milligrams daily that were prescribed by her treating physician. The patient had a past anesthetic history of spinal anesthesia for a cesarean section twenty years before the time of presentation. The patient history was otherwise unremarkable. Physical examination was only remarkable for uncontrolled blood pressure of 180/90 mmHg which was attributed to poor compliance with antihypertensive therapy. Her abdominal ultrasound was only significant for cholelithiasis. The patient was advised for better compliance to antihypertensive therapy and another anesthesia clinic visit was scheduled. The patient was seen again in the anesthesia clinic three months later. Her blood pressure at that time was found to be 150/80 mmHg, and the remainder of her examination was normal. Screening echocardiography recommended by the treating physician two months before that visit showed normal ejection fraction (66%) and mild tricuspid regurgitation in an otherwise normal study. Electrocardiogram (ECG) was performed before the anesthesia clinic visit and was normal (Figure 1). The patient was scheduled for surgery and admitted for the operation on the following day. Preoperatively, she was assessed by the anesthesia team in the hospital on the morning of the operation and her blood pressure was found to be 180/110 mmHg, which was again attributed to poor compliance with medication regimen. As the operation was scheduled on elective basis, it was therefore postponed until a tighter control of blood pressure could be achieved and the patient was discharged. She was seen four weeks later in the anesthesia clinic; there were no new complaints and there was no change in her examination except for her blood pressure, which was controlled, being 140/80 mmHg after proper compliance to her medication regimen. She was deemed fit for surgery, readmitted to the hospital, and scheduled for the operation as an American Society of Anesthesiologists physical status classification-2 (ASA-2) on the following day.

After admission, the patient's blood pressure was continuously monitored. Her routine labs, including blood electrolyte, were within normal limits. Premedication with 15 milligrams of oral midazolam was administered in two divided doses, once at bedtime and the second dose one hour before transfer to the operation theatre. On arrival to the theatre, her blood pressure (BP) and heart rate (HR) were 130/80 mmHg and 82 beats per minute (bpm), respectively, and her peripheral oxygen saturation (SpO_2_) was 100%. The patient had a 20-gauge cannula fixed in the surgical ward; another 18-gauge cannula was fixed in the operation theatre before induction. Intravenous infusion of lactated ringer was started; a total of around 350 ml of lactated ringer was transfused before induction. Standard monitoring of HR, BP, SpO_2_, and ETCO_2_ was connected. Invasive blood pressure monitoring and bispectral index monitoring were not connected as they are not part of the routine monitoring in the institution. Induction of general anesthesia was achieved using an intravenous injection of 150 milligrams of propofol and 10 micrograms of sufentanyl. Muscle relaxation was achieved using 8 milligrams of intravenous cisatracurium. The patient was intubated using a size 7 endotracheal tube; she was then connected to mechanical ventilation using volume control mode with a tidal volume of 450 ml, respiratory rate of 12 per minute, and positive end-expiratory pressure (PEEP) of 5 mmHg. End-tidal carbon dioxide (ETCO_2_) was maintained at about 38 mmHg and peak inspiratory pressure (PIP) was maintained at 15 centimeters of water (cmH_2_O). Anesthesia was maintained with 45% oxygen mixture with air and inhalation of 6% desflurane. Almost ten to twelve minutes after induction and preparation, the surgical team proceeded with Veress needle insertion and peritoneal insufflation. Insufflation was performed using carbon dioxide (CO_2_) at a flow rate of 40 liters per minute (L/M) to achieve an intraperitoneal pressure of 20 mmHg. Immediately after starting peritoneal insufflation, the patient developed sinus bradycardia and her heart rate started to drop reaching 35 to 40 bpm within a few seconds; however, there was no change in her ETCO_2_ or SpO_2_. The anesthetist immediately instructed the surgeon to stop the insufflation and deflate the abdomen. Intravenous injection of atropine (1 mg), which was prepared preoperatively, was given immediately and flushed with intravenous saline. Blood pressure reading obtained at the time did not show change in blood pressure. While the atropine was being administered, the patient's heart rate continued to drop, reaching 10–15 bpm, while her ETCO_2_ did not change. It was then decided that the patient would progress into cardiac arrest, so the anesthetist called for help of senior anesthesia staff to be present in the operating room and ordered the assistant staff to prepare intravenous adrenaline. The anesthetist then could not palpate the patient's pulse, so cardiopulmonary resuscitation was started. The anesthetist started chest compressions and an injection of adrenaline (100 micrograms) was given intravenously. The patient's pulse was restored within 30 seconds as sinus tachycardia of 124 bpm. Her blood pressure was 130/90 mmHg, SpO_2_ was 99%, and ETCO_2_ was 32 mmHg. An arterial line was then fixed under ultrasound guidance to monitor blood pressure, and blood samples were taken to check for blood electrolytes, gases levels, and cardiac markers, including troponin and creatine kinase-MB (CK-MB). During the following 15 minutes, the patient's vitals were closely monitored as the case was being discussed between the seniors of the surgical and anesthesia teams. During that time, the results of the blood gases and electrolytes came back and were within normal limits; in addition, there were no changes in the patient's ECG, except for sinus tachycardia that gradually returned to normal sinus rhythm. It was noted by the team that the insufflation flow rate had been high, and it was assumed that this was the cause of the event.

After being reassured that the patient was vitally stable and that there were no signs of cardiac or metabolic insult, the case was deemed fit to proceed for surgery. The surgical team started the operation again approximately 30 minutes after the bradycardia event. Another two milligrams of cisatracurium was administered, and a total of around 750 ml of lactated ringer was transfused by that time. The second time, peritoneal insufflation was performed using CO_2_ at a flow rate of 8 liters per minute (L/M) to achieve intraperitoneal pressure of 12 mmHg; pneumoperitoneum was achieved without complications. The patient was positioned in the reverse Trendelenburg left tilt position and the surgery was completed uneventfully within 20 minutes. The abdomen was deflated with the patient in the supine position. Intravenous injection of 5 milligrams of morphine was given and then the patient was extubated smoothly and shifted to the postanesthesia care unit (PACU). In the PACU, the patient was kept in the semi-sitting position and her vital signs and conscious level were closely observed. She was assessed for any postoperative pain or any new complains, and 1 gram of acetaminophen was given for pain control. The result for the confirmatory cardiac marker levels came back by that time and was within normal limits.

**Figure 2:**
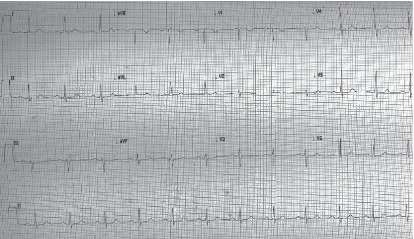
Postoperative ECG performed upon discharge from the PACU.

The patient was discharged from the PACU to the surgical ward after around 30 minutes with an Aldrete score of 10. Follow-up ECG (Figure 2) was performed and troponin levels were examined postoperatively in the surgical ward, both were within normal limits. The patient was seen in the surgical ward postoperatively by the anesthesia team after being discharged and then in the evening and then again on the next morning; on all occasions, she was vitally stable and fully conscious, with no complains. The patient was assessed by a cardiologist before discharge and was deemed free from cardiovascular problems. The patient was counselled about the intraoperative events by the surgical and anesthesia teams and was discharged on her postoperative day 3. She was scheduled for a routine follow-up visit to the surgical outpatient clinic one week following discharge. She was seen in the outpatient clinic after one week and was stable vitally and had no complains.

## 3. Discussion

Bradycardia progressing into cardiac arrest has been recognized as a complication of laparoscopy since its development [15, 16]. Many factors related to peritoneal insufflation have direct effects on the cardiovascular system. Peritoneal stretch due to insufflation leads to increase in vagal tone [12]. Vagal bradycardia has been reported in many gynecological and general surgical cases. In gynecological cases, the onset of bradycardia was related to direct stretch and manipulation of the peritoneum; in other cases, it was related to the rate of CO_2_ insufflation [14, 17, 18]. The high flow rate of CO_2_ leads to rapid peritoneal stretch, causing a strong and fast vagal response [17]. Several precautions have been implemented to prevent this from happening, including limiting the filling pressure of the peritoneum to 15 mmHg [19]. However, it has been noted in previous reports that even when the pressure is limited, the high flow of CO_2_ will inevitably cause rapid peritoneal stretch as the volume inflated will increase [17]. This can be overcome by decreasing the insufflation flow rate to slowly achieve the desired intraperitoneal pressure to facilitate the surgery [17, 20]. As of now, there is no clear recommendation regarding the optimal flow rate during insufflation to avoid such events, and the recommendation is only focusing on limiting the intra-abdominal pressure to 12–15 mmHg [21]. In our patient, the rate of CO_2_ insufflation was very high at the first time, at 40 L/M. We suspected that this was the direct cause of the event and it was confirmed later after the patient recovery, with the absence of recurrence of bradycardia with slow insufflation rate. After discussion with the surgical team, we gave a recommendation that during all future laparoscopic surgeries, the initial rate of peritoneal gas flow should not exceed 15 L/M.

Premedication with vagolytic agents has been shown to blunt the effect of peritoneal stretch on heart rate. The incidence of bradycardia was found to be lower in cases where muscle relaxants with vagolytic activity were used [13, 22]. It is, however, not commonly recommended to use anticholinergic medications preoperatively to prevent bradycardia, as studies have failed to show significant benefit in terms of mortality and morbidity [23, 24]. We did not use anticholinergic medications with our patient preoperatively, as there was no indication for it as mentioned above. In addition, we used cisatracurium for muscle relaxation, which does not have a clear vagolytic activity [25]; thus, we assume that the vagal response could not have been blocked by preoperative medications.

The risk of developing bradycardia or cardiac arrest intraoperatively increases with coexisting comorbidities [13]. Cardiovascular disease and the use of antihypertensive medications could exacerbate the effect of vagal bradycardia. Medications that lower the heart rate, such as beta blockers, might also increase the risk of developing intraoperative bradyarrhythmia progressing into cardiac arrest [10, 20]. The absence of cardiac disease, however, does not eliminate the risk, as bradyarrhythmia was reported in 5–25% of healthy young patients undergoing laparoscopy [11, 12, 18, 26]. The fact that our patient was taking bisoprolol preoperatively could have increased her risk of cardiovascular complications during the surgery.

It is important to consider the effect of anesthetic agents on the patient's cardiac status. Bradycardia commonly occurs after administration of propofol and sufentanyl and rarely after administration of cisatracurium [27, 28]. In our patient, the heart rate did decrease after induction, but it did not go below 60 bpm. Additionally, almost twelve minutes have elapsed after induction when the patient started to deteriorate. Although the anesthetic agents could potentiate the vagal response, it cannot be claimed that they alone were the cause of such event [29].

It is important to consider other causes that could have happened in our case for her pre-arrest. Other factors related to laparoscopy, including gas embolism, decreased preload, pneumothorax, and vascular injury should be considered in similar cases. Gas embolism occurs when the insufflated CO_2_ dissolves into the venous or lymphatic circulation and then into the lungs. The characteristic features of gas embolism include decreased ETCO_2_, tachycardia, and decreased blood pressure [13, 30]. None of these signs were present in our patient, so it is unlikely that there was gas embolism in our case. Patients can develop decrease in cardiac output during laparoscopy due to gas compression on the inferior vena cava which decreases the preload [10]. It is unlikely that this has happened in our case, as decreased preload would be accompanied by tachycardia rather than bradycardia. Direct vascular injury by the Veress needle or the laparoscopic trocars can cause significant hemorrhage that could progress into cardiac arrest [9, 18]. In our case, the patient did not show signs of volume depletion, such as hypotension or tachycardia; additionally, there was no evidence of vascular injury at the second time when the abdomen was visualized. It is also unlikely that pneumothorax could have happened in our case as there were no signs of desaturation or tachycardia.

In some cases of cardiac arrest during laparoscopy, the actual cause might not be identified [16, 31]. The initial steps in the management should be implemented irrespective of the cause. These steps include resuscitation following the Advanced Cardiac Life Support (ACLS) protocol [32]. In the cases reported previously in the literature, management of bradycardia included cessation of abdominal insufflation, deflation of the abdomen, and administration of atropine [11, 17]. These were the steps we followed in our patient. It is not recommended to start the cardiopulmonary resuscitation (CPR) until confirming that the patient has no pulse. In our patient, although there was an electrical activity detected by the ECG as severe sinus bradycardia, the anesthetist was unable to palpate the pulse, so CPR was started early and immediately after administering atropine. In addition, adrenaline was administered prematurely during the first minute of CPR. We cannot claim that what we did was the ideal management for such cases, but the CPR and adrenaline administration were preemptive measures taken to prevent the patient from going into asystole. The proper management would be to administer adrenaline only if atropine administration was ineffective [32]. Placement of an arterial line to monitor blood pressure continuously was crucial after such a devastating event. Additionally, assessing the cardiac and metabolic status through blood investigation was necessary before taking any further decision regarding the progress of the surgery or the disposition of the patient. After identifying and correcting the cause of the insult, and confirming that the patient was stable, we took the decision to proceed with the surgery. We believed that it would be in the best interest of the patient not to abort and reschedule, as subjecting the patient to anesthesia and laparoscopy a second time could have increased the risk of morbidity and mortality. It was also crucial that the patient be followed up after disposition to monitor for any sequela of the event. Most of the cases reported in the literature recovered without significant complications and had a routine postoperative course [9, 11, 17]. Our patient was discharged after all involved teams, including surgeons, anesthetists, and physicians, reassured that she was stable and fit for home follow-up, as should always be the case in such patients.

## 4. Conclusion

In conclusion, bradycardia and subsequent cardiac arrest are not uncommon during laparoscopy. Vagal response to rapid peritoneal stretch is usually the main cause, and it can happen due to high flow rate of gas insufflation. The management consists of cessation of gas insufflation, deflating the abdomen, and administering atropine. Supportive management should be performed accordingly, and the patient should be followed closely afterwards, although most patients recover without significant harm.

## Data Availability

Data and materials related to the case are available upon request from the corresponding author.

## Abbreviations

ASA: American Society of Anesthesiologists physical status classification
ACLS: Advanced cardiac life support
BP: Blood pressure
bpm: Beats per minute
CK-MB: Creatine kinase-MB
cmH_2_O: Centimeters of water
CO_2_: Carbon dioxide
ECG: Electrocardiogram
ETCO_2_: End-tidal carbon dioxide
HR: Heart rate
L/M: Liters per minute
mg: Milligram
mmHg: Millimeters of mercury
PACU: Postanesthesia care unit
PEEP: Positive end-expiratory pressure
PIP: Peak inspiratory pressure
SpO_2_: Peripheral oxygen saturation.
